# A Rare Case of Primary Intracerebral Malignant Melanoma

**DOI:** 10.7759/cureus.43359

**Published:** 2023-08-12

**Authors:** Quratulain Khan, Sana Akhtar, Waleed Khalid, Anis Rehman, Kashif Siddique

**Affiliations:** 1 Radiology, Shaukat Khanum Memorial Cancer Hospital and Research Centre, Lahore, PAK

**Keywords:** neural crest cells, melanocytes, malignant, intracranial, melanoma

## Abstract

Malignant melanomas occurring in the central nervous system are mostly metastatic. Primary intracranial malignant melanomas are a rare entity, accounting for 0.07% of all brain tumors. In the central nervous system, melanocytes originate from the neural crest cells that are found in the leptomeninges. Only a few cases of malignant melanoma primarily arising from the brain have been reported in the literature to date.

We report a rare case of primary intracerebral malignant melanoma in a 39-year-old female. Through this case report, our aim is to highlight the role of imaging in the early diagnosis and management of malignant melanoma.

## Introduction

Most malignant melanomas of the central nervous system are metastatic, constituting 12-17% of all intracranial malignancies. Primary intracranial malignant melanomas are rare entities. These aggressive primary melanomas account for 0.07% of all brain tumors [1-2].

Malignant melanomas arise from the melanocytic cells that are mostly present in the uvea, cerebral parenchyma, leptomeninges, mucous membranes, and skin [3]. In the central nervous system, melanocytes originate from the neural crest cells that are found in the leptomeninges, making them the most common intracranial site for primary tumors [3].

Prognosis of primary intracranial malignant melanoma is variable depending on the tumor site, the accuracy of the diagnosis, and the extent of tumor growth. The presenting symptoms of patients with primary intracranial malignant melanoma include raised intracranial pressure (43%), a neurological deficit (35%), or subarachnoid hemorrhage (16%) [4].

In our case report, we will discuss the clinical presentation and radiological findings of primary intracerebral malignant melanoma arising from the left insular cortex.

## Case presentation

**Figure 1:**
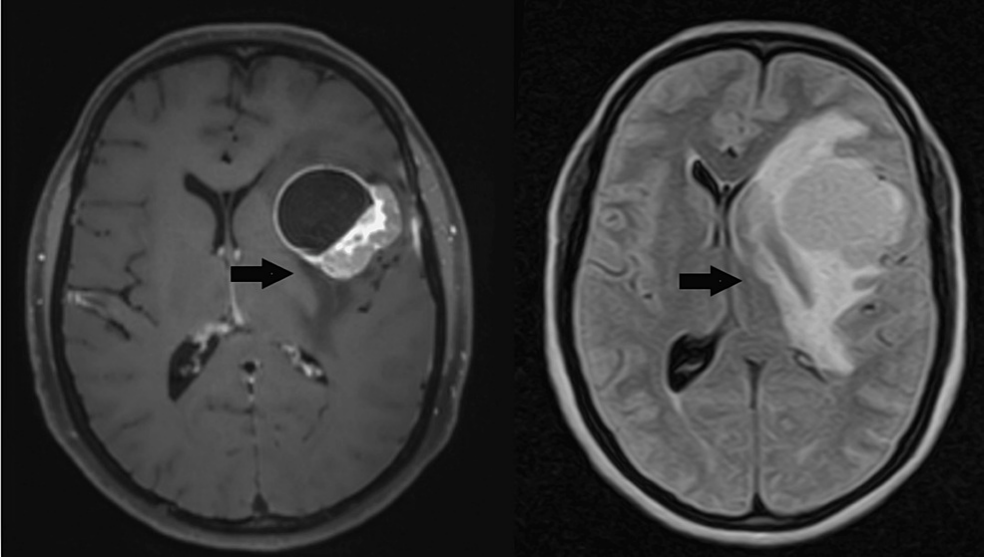
An axial post-contrast T1 image shows a large mass in the left insular region, demonstrating peripheral rind contrast enhancement with FLAIR hyperintense signals in the left anterior temporal lobe, left basal ganglia, and left frontal lobe (black arrows). FLAIR:  fluid attenuated inversion recovery

A 39-year-old female presented to our hospital with complaints of headaches for the last two years that had worsened in the last two months. The headache was associated with numbness on the left side and epistaxis for two days. There was no history of prior surgery. On thorough clinical examination, no visible cutaneous lesion was identified. The patient then underwent a contrast-enhanced brain MRI that showed a 4.1 x 4.2 x 3.8 cm mass in the left insular region, demonstrating a peripheral rind of thick nodular contrast enhancement along with vasogenic edema and a midline shift of 15 mm. The mass was encasing the left middle cerebral artery. Fluid attenuated inversion recovery (FLAIR) hyperintense signals were also seen involving the left anterior temporal lobe, left basal ganglia, left insular region, and left frontal lobe (Figure 1).

**Figure 2:**
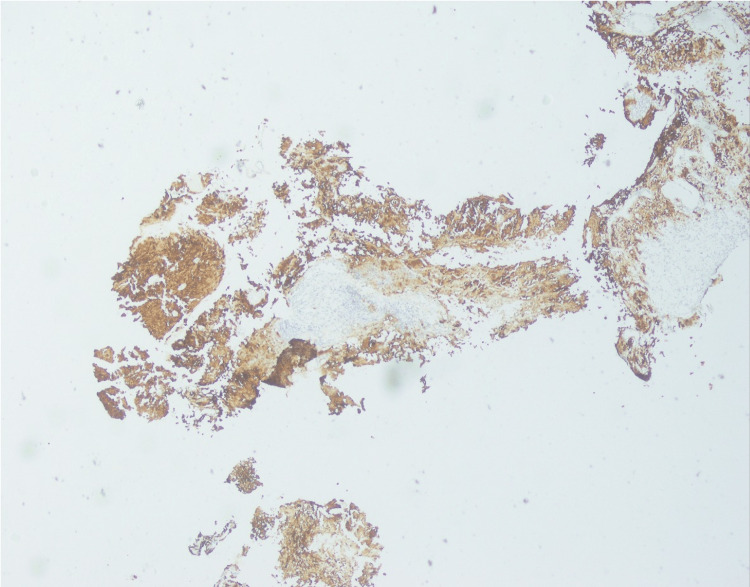
Histopathology results reveal a tumor with melanocytic differentiation.

Subsequently, a stealth-navigated left temporal craniotomy and biopsy of the tumor were performed. During the surgery, a firm vascular tumor was seen, and clinical suspicion of glioma vs. metastasis was raised. However, the biopsy results revealed a tumor with melanocytic differentiation (Figure 2).

Differential diagnoses of primary melanoma and clear cell sarcoma had been proposed. Further, Fluorescence in situ hybridization (FISH) for the EWSR1 gene was done and turned out to be negative. Human melanoma black-45 (HMB-45), Melan A, and SRY-box transcription factor 10 (SOX 10) stains were positive. So the final diagnosis of primary intracranial malignant melanoma was made.

**Figure 3:**
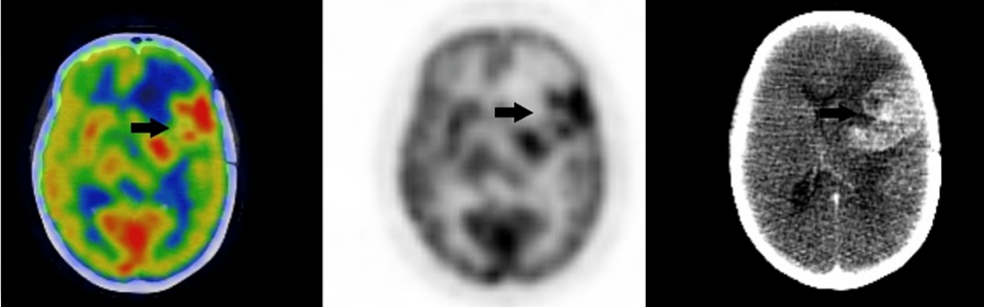
Enhancing hypermetabolic mass in the left frontal-parietal region with vasogenic edema and contralateral midline shift (black arrows).

**Figure 4:**
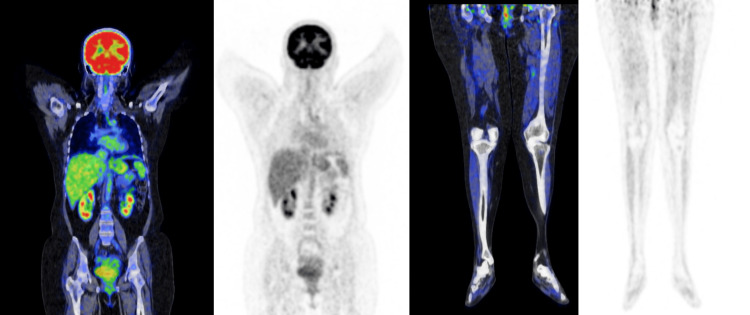
A whole-body FDG-18 PET/CT scan was negative for any other possible site of melanoma. FDG-18 PET/CT: ^18^fluorodeoxyglucose (FDG) positron emission tomography/computerized tomography

Fluorodeoxyglucose (FDG)-positron emission tomography/computerized tomography (PET/CT) was performed after a craniotomy and biopsy that demonstrated hypermetabolic left frontal-parietal region mass. The rest of the scan was negative for abnormal FDG uptake. No bowel-related or cutaneous mass was noted (Figures 3-4).

A limitation of our case report is not excluding a bowel melanoma lesion by endoscopy. However, positron emission tomography (PET) is a sensitive examination, and no abnormal activity was noted.

**Figure 5:**
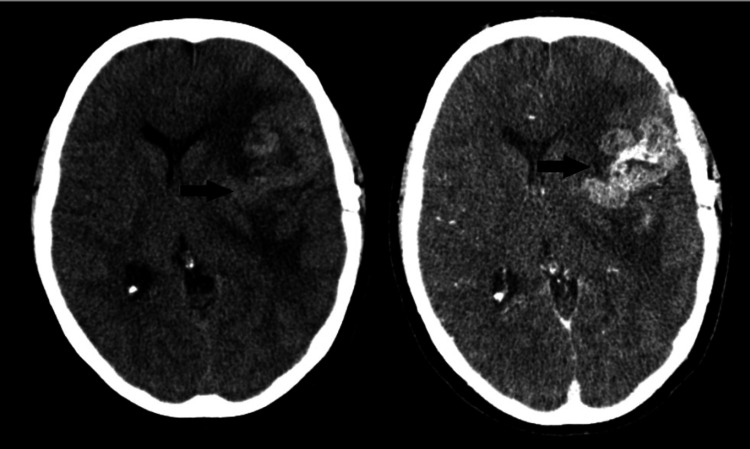
An unenhanced and enhanced CT scan of the brain demonstrates post-left parietal approach craniotomy changes with an enhanced hyperdense mass centered over the left insular cortex (black arrows).

The post-craniotomy CT scan of the brain also demonstrated heterogeneously enhanced mass with surrounding vasogenic edema involving the left frontal-parietal region and persistent midline shift (Figure 5).

During the course of her admission, our patient developed diffuse cerebral edema and raised intracranial pressure. Mannitol infusions were given but the Glasgow Coma Scale (GCS) kept on lowering. Three fractions of 60 Grey' whole-brain radiotherapy were given along with intravenous dexamethasone (4 mg) during the hospital stay; a 0.5 mg oral dose and four tablets twice a day were prescribed upon discharge. Despite treatment with radiotherapy and steroids, our patient could not survive and passed away after five months due to cerebral edema and raised intracranial pressure before any systemic treatment could be given.

## Discussion

Melanoma originates from the melanocytes and can occur possibly anywhere in the human body. Melanocytes are predominantly found in the skin, mucous membranes, leptomeninges, cerebral parenchyma, and uvea [5]. Primary intracranial melanomas most commonly arise from the leptomeninges, accounting for 0.07% of all brain tumors [6-7]. The contrasting features of primary cerebral melanoma from metastatic melanoma include early age of presentation (usually before 50 years of age) and uncommon systemic spread of the former [8]. Primary intracranial melanomas are further classified into four different categories: melanosis of the brain or spinal cord meninges associated with cutaneous pigmentations (phacomatoses or neurocutaneous melanoses), primary cerebral isolated melanoma, discrete spinal cord melanoma, and diffuse leptomeningeal melanomatoses [9]. Our patient had a cerebral tumor without evidence of leptomeningeal or systemic involvement. Therefore, the patient was considered to have primary cerebral isolated melanoma.

Primary cerebral melanomas pose difficulty in preoperative diagnosis. The imaging features are also variable. On a CT scan, typical primary cerebral melanomas are hyperdense with intense post-contrast enhancement, like in our case. Intratumoral hemorrhage and melanin are the usual causes of increased attenuation on a precontrast CT scan [10]. However, these can also be iso- to hypodense in attenuation due to old clotted blood [11]. Similarly, the MRI features are also variable based on the melanoma type and intratumoral hemorrhage. In typical melanotic melanomas, paramagnetic effects are depicted on the T1 weighted image (T1WI) and T2 weighted image (T2WI) MRI, mainly due to the presence of melanin. The paramagnetic property of melanin depends on the free radical formation of paramagnetic metals binding to melanin. There is a shortening of both T1 and T2 relaxation times, producing hyperintense signals on T1WI and hypointense signals on T2WI. Whereas, amelanotic melanoma and melanoma without a hemorrhagic component appear iso- to hypointense on T1WI and hyperintense on T2WI [12].

The mainstay of treatment for primary cerebral melanomas is complete surgical resection followed by postoperative radiotherapy [11-12]. Dacarbazine was previously considered the most effective chemotherapy drug, usually used after surgery or alongside radiotherapy. The effectiveness rate of this drug is approximately 16% to 20%. At present, combination regimens exist with a higher response rate and better central nervous system (CNS) penetration, including targeted immunotherapy. Some previous research suggests a positive role of stereotactic radiosurgery (SRS), alone or in conjunction with whole-brain radiotherapy, in improving the lifespan and quality of life of the affected patients [6]. Since our patient was symptomatic, her treatment was initiated with steroids and radiotherapy, but she could not survive.

## Conclusions

Primary malignant melanomas arising from the central nervous system are rare entities. They pose difficulty with a definitive diagnosis owing to their nonspecific presentation and imaging findings. The correlation between immunohistochemical staining and imaging findings provides the most definitive method to diagnose primary intracranial malignant melanomas and differentiate them from other tumors. The mainstay of treatment includes surgery and combination therapy with radiotherapy, chemotherapy, and immunotherapy. However, there is still room for improvements in targeted therapies to provide more efficient treatments for primary cerebral melanomas.
